# Prevalence and risk factors related to poor outcome of patients with severe *Plasmodium vivax* infection: a systematic review, meta-analysis, and analysis of case reports

**DOI:** 10.1186/s12879-020-05046-y

**Published:** 2020-05-24

**Authors:** Manas Kotepui, Kwuntida Uthaisar Kotepui, Giovanni De Jesus Milanez, Frederick Ramirez Masangkay

**Affiliations:** 1 Medical Technology, School of Allied Health Sciences, Walailak University, Thasala, Nakhon Si Thammarat Thailand; 2 Department of Medical Technology, Far Eastern University, Manila, Philippines

**Keywords:** *P. vivax*, Discharge, Treatment outcome, Severe malaria, Poor outcome

## Abstract

**Background:**

*Plasmodium vivax* rarely develops severe complications when compared to severe falciparum malaria. However, severe vivax malaria also needs urgent, intensive care and treatment as severe falciparum malaria. This systematic review aimed to explore pooled prevalence of severe vivax malaria and to identify factors related to poor outcome of patients who developed severe manifestation.

**Methods:**

The systematic review conducted by two reviewers independently through searching of research publications related to severe *P. vivax* malaria in three databases including MEDLINE, Web of Science (ISI), and Scopus until October, 22 2019. The pooled prevalence of severe vivax malaria was achieved using STATA and RevMan 5 Software. Factors related to poor outcome of patients with severe vivax malaria were analyzed using SPSS 11.5 Software.

**Results:**

Among 2615 research publications retrieved from three databases, 49 articles reporting on 42,325 severity cases were selected for calculating pooled prevalence. Seventy-six patients from case reports, case series, letter to editors, and research communications were collected to identify factors related to poor outcome of patients with severe vivax malaria. The results showed that severe anemia, jaundice, respiratory distress, impaired consciousness, and renal failure were the most common major manifestations of severe malaria guided by the World Health Organization (WHO) criterion. The meta-analysis indicated that severe malaria was less frequent in patient with *P. vivax* compared to those with *P. falciparum* (*P* -value < 0.00001, OR = 0.38, 95% CI = 0.25–0.56, I^2^ = 87%). In addition, thrombocytopenia, anemia, hepatitis, and severe thrombocytopenia were the most common minor complications. Analysis of cases indicated that convulsion, respiratory distress, renal failure, jaundice, anuria/oliguria, and complication during treatment impacted on longer hospital stays compared to other severe complications (*P*-value < 0.05). Respiratory distress was frequently found after first treatment with anti-malarial drugs (*P*-value = 0.002). Renal failure was frequently found before treatment with anti-malarial drugs (*P*-value = 0.016). Mean days of fever and higher pulse rates at presentation were predictors of poor outcome among patients with severe vivax malaria (*P*-value < 0.05).

**Conclusions:**

Severe anemia was the most common major manifestation of *P. vivax* malaria guided by the WHO criterion. Severe anemia was found less frequently in patients with *P. vivax* than those with *P. falciparum*. Renal failure, jaundice, anuria/oliguria, and complication during treatment along with, mean days of fever and higher pulse rates at presentation might be predictors of poor outcome of patients with severe vivax malaria.

## Background

*Plasmodium* spp. infection among humans were estimated at 228 million cases of malaria occurring worldwide in 2018, especially in the African Region (213 million or 93%), followed by the South-East Asia Region (3.4%) and the Eastern Mediterranean Region (2.1%) with an estimated 405,000 deaths [1]. *P. vivax* infection was manifested in the South-East Asia Region (53%), with the majority being in India (47%) [1]. *Plasmodium vivax* is one of five *Plasmodium* species infecting humans and is recognized as less severe than *Plasmodium falciparum* infection. However, severe vivax malaria is a medical emergency defined as one or more complications with the presence of *P. vivax* asexual parasitemia with no parasite density threshold [2]. The major complications of severe malaria as defined by the World Health Organization (WHO) 2014 included impaired consciousness, jaundice, pulmonary edema, acute renal failure, severe anemia, bleeding, acidosis, hyperparasitemia, respiratory distress, and hypoglycemia [2]. Any of these complications can develop rapidly and progress to death within hours or days. In recent years, a large number of case reports about severe malaria caused by *P. vivax* have been reported. Previous studies indicated that *P. vivax* infections can lead to severe malaria including renal failure [3–5], bleeding [6, 7], hyperparasitemia [8, 9], impaired consciousness [9–12], jaundice [6, 13, 14], prostration [15], pulmonary edema [16–18], respiratory distress [19–22], severe anemia [4, 14], and shock [23, 24].

This systematic review, meta-analysis, and analysis of cases aimed to highlight the prevalence of severe malaria severe complications caused by *P. vivax* malaria. The secondary objective was to identify factors related to poor outcome of patients who developed severe vivax malaria in literatures.

## Methods

### Search of articles from 3 published databases

This systematic review and meta-analysis was reported according to the PRISMA statement guidelines [25]. The searches were performed in the three published databases including MEDLINE, Web of Science (ISI), and Scopus. The searches focused on studies related to the prevalence and individual patient reports (case reports and also case series, correspondences, letter to editors) of severe vivax malaria, which published up to October 22, 2019. The search strategy used to retrieve relevant articles was the following “*Plasmodium vivax*”[All Fields] AND “complications”[All Fields]. EndNote Version X7 (Tomson Reuters, New York, NY) was used to manage references retrieved in this study.

### Eligibility criteria

All studies in the English language which reported prevalence, incidence, and individual case reports of severe vivax malaria infection were included. Exclusion criteria included any studies that reported animal studies, clinical drug trial/drug studies, experimental/investigation studies, placental/maternal malaria, *P. vivax* with no related topic/no complication, studies not about malaria prevalence, malaria studies but not on *P. vivax* infection, co-infection with other agents/with underlying diseases, reviews, and co-infection of other species of *Plasmodium* spp., which were excluded after screening the titles and abstracts. Only articles reporting on severe *P. vivax* mono-infection were selected. Studies were additionally excluded following full-text review if they reported incomplete prevalence on severe vivax complications.

### Data extraction

Two independent reviewers (Manas Kotepui and Kwuntida Uthaisar Kotepui) were responsible for searching and selecting articles associated with the inclusion criteria. First, Endnote X7 Software (Clarivate Analytics, Canada) was used to manage the references retrieved from three databases. Duplicate references among the three databases were excluded. The references were then being screened for their title and abstract. Screened articles were examined for full text evaluation. Any discrepancy between two reviewers were consulted with two independent reviewers (Giovanni De Jesus Milanez and Frederick Ramirez Masangkay) to determine whether articles can be included in the study. The articles that passed the inclusion and exclusion criteria were encoded into an Excel Spreadsheets (Microsoft, USA). Each article contained these data: author, year of publication, severity sign, age, gender, country of infection, fever, days of fever, signs and symptoms at presentation, pulse, respiratory rate, systolic blood pressure, diastolic blood pressure, spleen and liver abnormality, hemoglobin, leukocyte count, platelet count, stage of *P. vivax* found in blood film examination, Rapid diagnostic test (RDT) used in the study, time point of severity sign, complication after treatment, number of anti-malarial drugs given, discharged days, improving days, and days of parasite clearance.

### Criteria of severe malaria and treatment outcome

Criteria of severe vivax malaria was defined by the WHO Guidelines including impaired consciousness, convulsion, prostration, pulmonary edema, acute renal failure, severe anemia, bleeding, jaundice, acidosis, hyperparasitemia, respiratory distress, and hypoglycemia with no parasite density threshold [2]. Those complications defined by the WHO Guidelines were categorized as major complications, whereas other complications reported in literatures which were not defined by the WHO Guidelines, were reported as minor complications in the present study. Treatment outcome of severe malaria on case reports was based on the number of days discharged from hospital and also days of improving from severe manifestation. The number of discharged days was divided as follows: less than one week as a good outcome, whereas more than one week as a poor outcome.

### Meta-analysis

The pooled prevalence and 95% CI of complications among severe vivax malaria was undertaken to perform a meta-analysis with STATA Software Version 16 (Stata Corp LP, Texas USA). The pooled prevalence was calculated following a previous research [26] by using the the following command in STATA:

#### Metaprop case population, random

The prevalence of severe complications between *P. vivax* and *P. falciparum* was assessed using Cochrane Q (Chisquare) with Odds ratio, Moran’s I^2^ (Inconsistency), and subgroup analyses using RevMan 5 Software (Version 5, London, UK) [27]. A random-effects model was used to estimate the summary Mantel-Haenszel odds ratio of severe complications among patients infected with *P. vivax* and those with *P. falciparum*.

### Statistical analysis

In order to identify factors related to poor outcome of severe vivax malaria, individual patient’s data from all case reports, case series, letter to editors, and correspondences were input to SPSS 11.5 Statistical Software (SPSS Inc., Chicago, USA)**.** Qualitative demographic data (e.g., gender, nationality, severity sign, country of infection, clinical and laboratory parameters time point of severe manifestation, treatment outcome, etc.) were analyzed using the frequency, percentage, and Chi-squared test with 95%CI. Differences among quantitative variables such as age, temperature, systolic blood pressure, diastolic blood pressure, pulse rate, days of fever, hemoglobin, leukocyte count, and platelet count between two groups were analyzed by an independent sample T test or Mann Whitney U test upon the distribution of the data.

## Results

### Search results

**Fig. 1:**
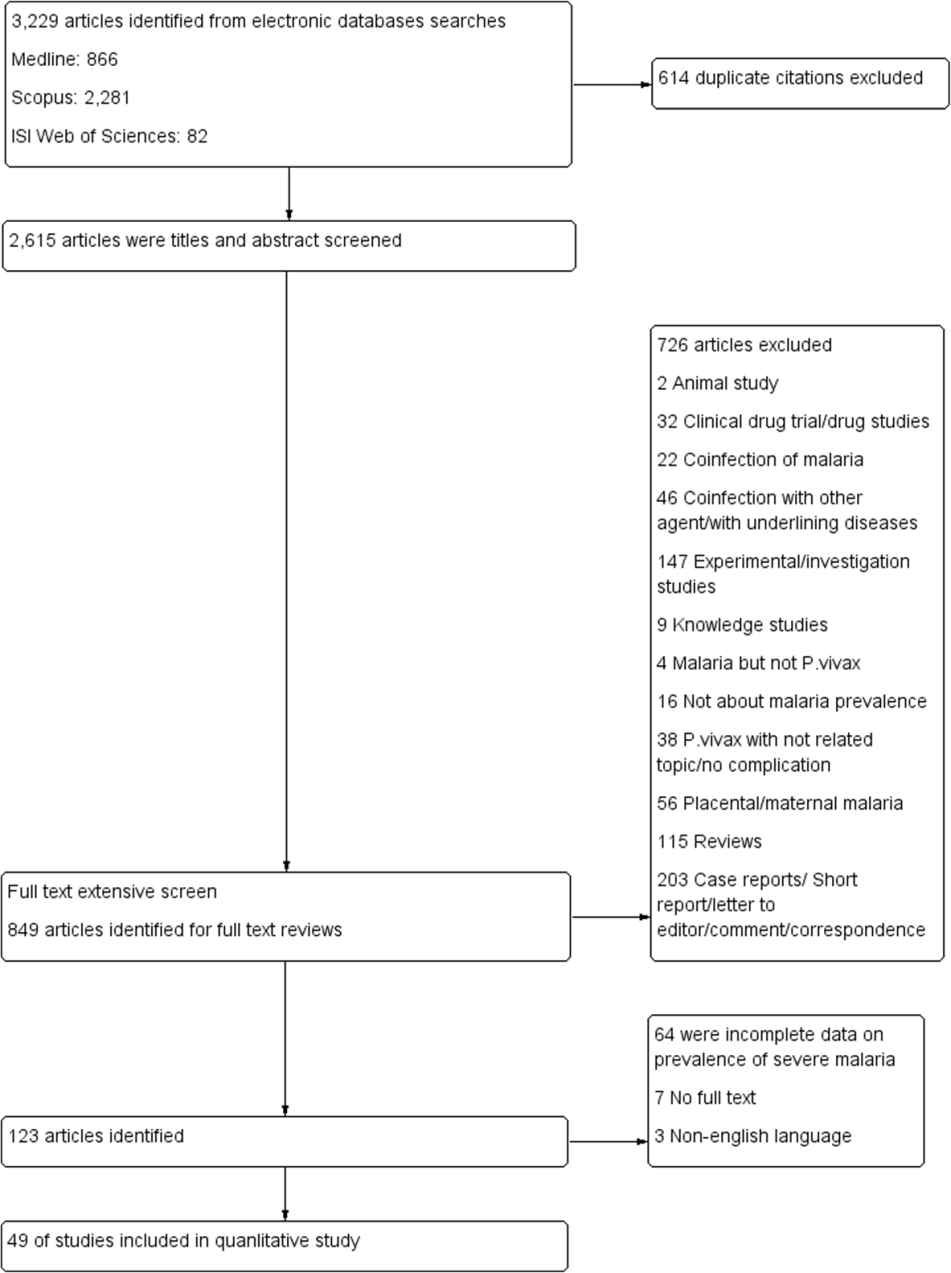
PRISMA diagram. Flow chart for study selection

All 3229 research articles were retrieved from three databases (866 from MEDLINE, 2281 from Scopus, and 82 from Web of Science). After deleting duplicate references, the titles and abstracts of 2615 articles were screened. Out of 849 articles that were selected for full-text screening, 123 articles were included for further review. Out of these 123 articles, 49 articles reporting prevalence of severe vivax malaria for calculating pooled prevalence were further selected and included for pooled analysis. In addition, out of 203 individual patient reports (from case reports and also case series, correspondences, letter to editors), 76 individual cases reporting on the prevalence of severity signs were selected based on the criteria for severe vivax malaria and used to identify factors related to poor outcome of severe vivax malaria in our study (Fig. 1).

### Characteristics of included studies

#### Articles for calculating pooled prevalence and 95%CI

**Fig. 2:**
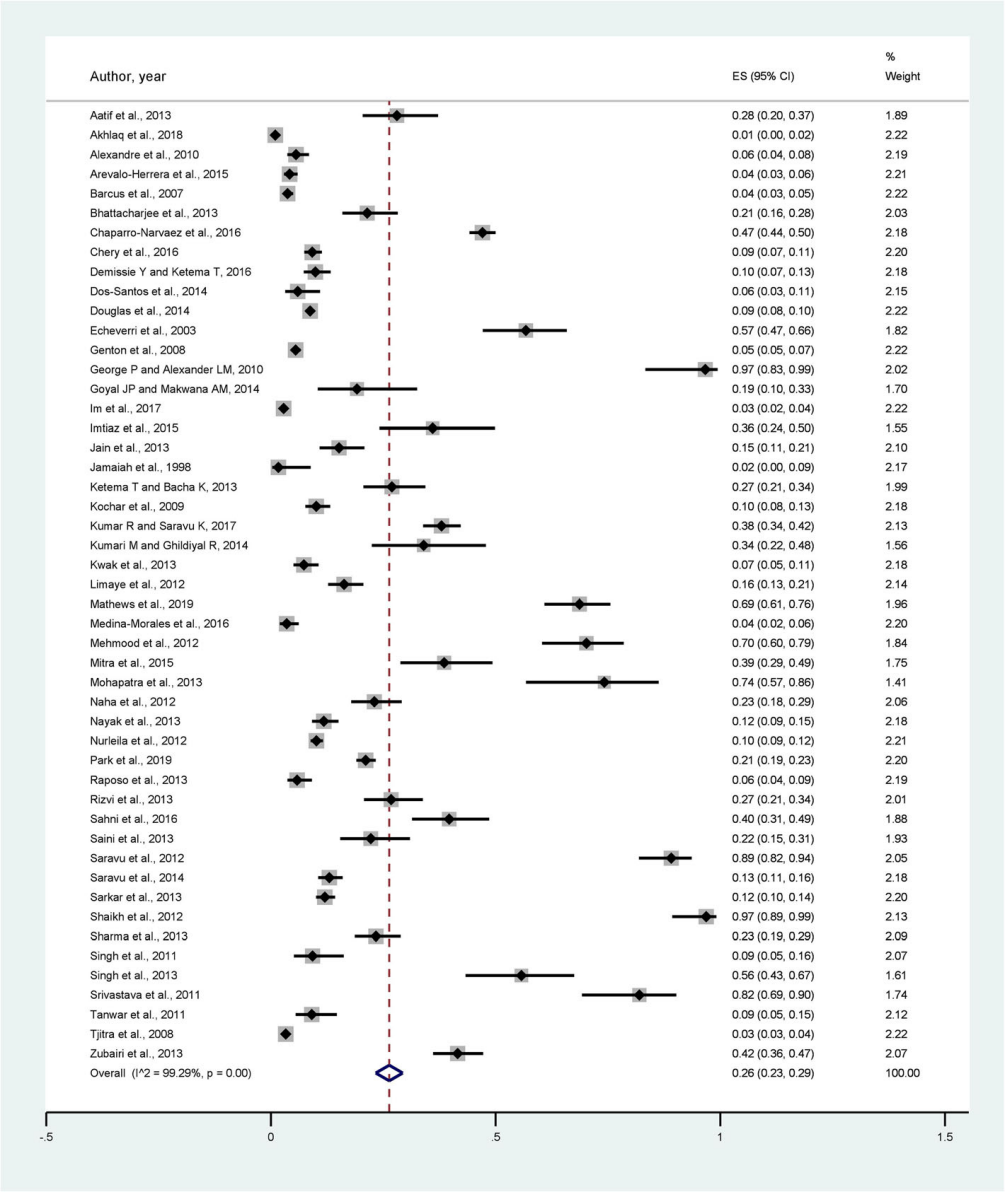
Clinical characteristics of 49 prevalence studies and prevalence rates

Characteristics of the 49 studies included in this study are listed in Table 1 and Fig. 2. Most of the studies reported severity signs of *P. vivax* malaria infection in 2013 (14, 28.6%), following by 2012 (6, 12.2%), 2014 (5, 10.2%), and 2016 (5, 10.2%). Most of the study’ participants were from India (25, 51%), Pakistan (6, 12.2%), and Indonesia (5, 10.2%).

**Table 1:** Clinical characteristic of included studies (49 prevalence studies)

Major complication (WHO, 2014)	Total patients with severity sign	Severity rate (%) Total ***P.vivax***, 42,325 cases
Respiratory distress	566	1.34
Acidosis	44	0.10
Pulmonary edema	126	0.30
Death	152	0.36
Cerebral malaria	471	1.11
Convulsion	75	0.18
ARF	355	0.84
Prostration	26	0.06
Hypotension/shock	267	0.63
Jaundice	634	1.50
Severe anemia	1001	2.37
Bleeding/DIC	242	0.57
Hyperparasitemia	31	0.07
Hypoglycemia	65	0.15
**Minor complication**		
Hyperpyrexia	57	0.13
Severe vomiting	139	0.33
Drowsiness	123	0.29
Hepatitis	308	0.73
Respiratory (NS*)	1	0.00
Severe vomiting	1	0.00
Sudden cardiac arrest	7	0.02
Hemoglobinuria/Hematuria	143	0.34
Anemia	1008	2.38
Thrombocytopenia	1243	2.94

*NS Not specified

#### Articles for identifying factors related to poor outcome of severe vivax malaria

Characteristics of the 76 studies with individual patient’ reports were listed in Table 2. All studies reported severe vivax malaria during 1993–2019. Most of the reports were published in 2010 (17, 22.2%), 2013 (11, 14.5%), and 2016 (6, 7.9%). Most of the major severe complications among reports were renal impairment (28, 36.8%), respiratory distress (22, 28.9%), and impaired consciousness (21, 27.6%).

**Table 2:** Clinical characteristics of 76 patients (from case reports)

Clinical Characteristics	Frequency (percent)
Year of publication	
- ≤ 2010	38 (50)
- > 2010	38 (50)
Age, mean (SD)	30.1 (11.5)
Impaired consciousness	
- Yes	21 (27.6)
- No	25 (22.4)
Pulmonary edema	
- Yes	3 (3.9)
- No	73 (96.1)
Renal failure	
- Yes	28 (36.8)
- No	48 (63.2)
Severe anemia	
- Yes	8 (10.5)
- No	68 (89.5)
Bleeding	
- Yes	8 (10.5)
- No	68 (89.5)
Jaundice	
- Yes	19 (25)
- No	57 (75)
Acidosis	
- Yes	4 (5.3)
- No	72 (94.7)
Hyperparasitemia	
- Yes	2 (2.6)
- No	74 (97.4)
Respiratory distress	
- Yes	22 (28.9)
- No	54 (71.1)
Convulsion	
- Yes	8 (10.5)
- No	68 (89.5)
Prostration	
- Yes	1 (1.3)
- No	75 (98.7)
Gender	
- Male	48 (64)
- Female	27 (36)
Country of infection	
- India	59 (77.6)
- Non-India	17 (22.4)
Days of fever	5.71 (2.58)
Cough	
- Yes	6 (7.9)
- No	70 (92.1)
Weakness	
- Yes	9 (11.8)
- No	67 (88.2)
Headache	
- Yes	12 (15.8)
- No	64 (84.2)
Malaise	
- Yes	3 (3.9)
- No	73 (96.1)
Rash	
- Yes	7 (9.2)
- No	69 (90.8)
Urine output abnormality	
- Yes	19 (25)
- No	57 (75)
Chills	
- Yes	36 (47.4)
- No	40 (52.6)
Rigors	
- Yes	27 (35.5)
- No	49 (64.5)
Abdominal pain	
- Yes	15 (19.7)
- No	61 (80.3)
Nausea	
- Yes	6 (7.9)
- No	70 (92.1)
Vomiting	
- Yes	15 (19.7)
- No	61 (80.3)
Edema	
- Yes	1 (1.3)
- No	75 (98.7)
Temperature, mean (SD)	38.5 (1.04)
Systolic blood pressure, mean (SD)	107.6 (24.5)
Diastolic blood pressure, mean (SD)	69 (15.9)
Pulse rate, mean (SD)	110.9 (19.9)
Respiratory rate, mean (SD)	30.3 (11.5)
Splenomegaly	
- Yes	23 (30.3)
- No	53 (69.7)
Hepatomegaly	
- Yes	17 (22.4)
- No	59 (77.6)
Hemoglobin, mean (SD)	9.47 (2.79)
Leukocyte, mean (SD)	6237.8 (2722.2)
Platelet, mean (SD)	85,360 (74,296.5)
RDT	
- Yes	43 (56.6)
- No	33 (43.4)
Time point of severe complications	
- Before treatment (at presentation)	50 (65.8)
- After treatment (with anti-malarial drug)	16 (21.1)
- Before and after treatment	10 (13.2)
Complication during treatment	
- Yes	34 (44.7)
- No	42 (55.3)
Second anti-malarial drug given	
- Yes	13 (17.1)
- No	63 (82.9)
Discharge/recovery/parasite clearance	
- > 7 days	41 (65.1)
- ≤ 7 days	22 (34.9)

### Prevalence of severe vivax malaria

**Fig. 3:**
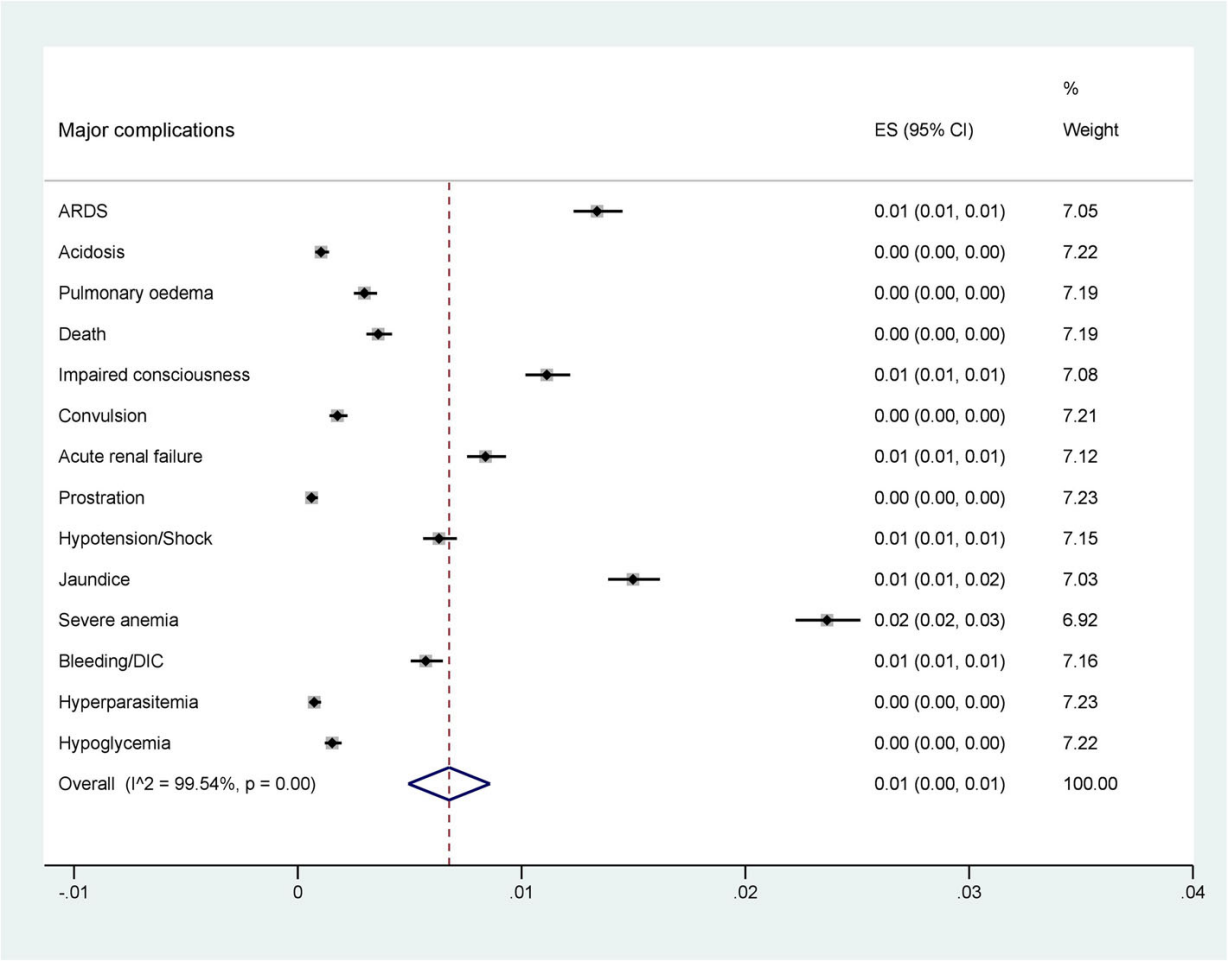
Pooled prevalence of severe complications (WHO, 2014)

**Fig. 4:**
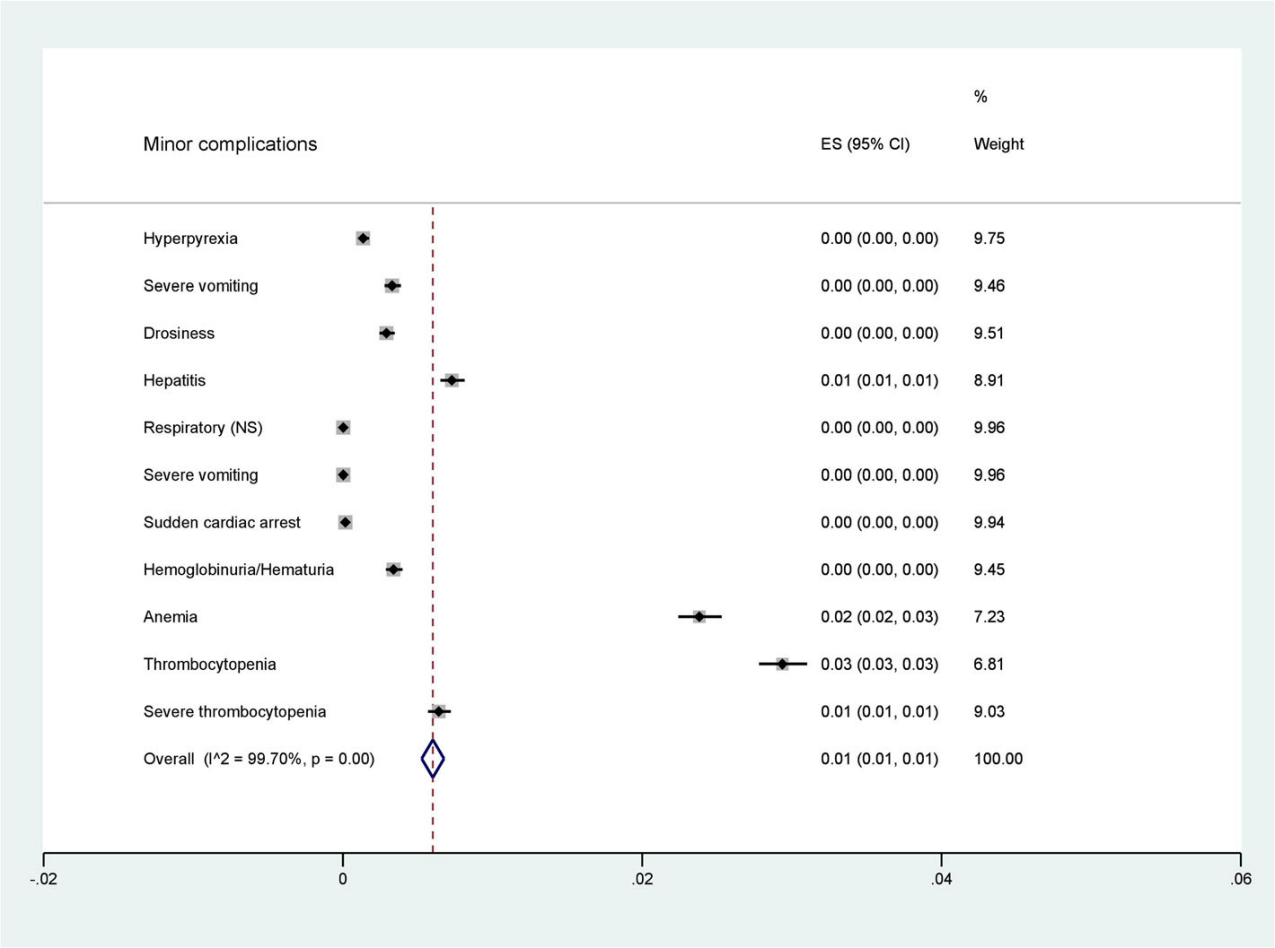
Pooled prevalence of minor complications

Among 49 studies reporting on 42,325 severity cases, 21 reported impaired consciousness, 1 prostration, 8 convulsion, 4 acidosis, 22 respiratory distress, 8 severe anemia, 28 renal failure, 19 jaundice, 3 pulmonary edema, 8 bleeding, 6 shock, and 2 studies reported hyperparasitemia. The prevalence of severe complications of each study is shown in Fig. 2. The pooled prevalence of each severe manifestation according to the WHO criteria is shown in Fig. 3. Moreover, the pooled prevalence of each minor complication including thrombocytopenia, severe thrombocytopenia, hyperpyrexia, severe vomiting, drossiness, hepatitis, sudden cardiac arrest, hemoglobinuria/hematuria, and moderate anemia is shown in Fig. 4.

Among these studies, the pooled prevalence of the five most commonly reported major complications were: severe anemia 0.02% (95% CI: 0.02, 0.03), jaundice 0.01% (95% CI: 0.01, 0.02), respiratory distress 0.01% (95% CI: 0.01, 0.01), impaired consciousness 0.01% (95% CI: 0.01, 0.01), and acute renal failure 0.01% (95% CI: 0.01, 0.01) (Fig. 3) (Data set 1). In addition, the pooled prevalence of the three most commonly reported minor complications were: thrombocytopenia 0.03% (95% CI: 0.03, 0.03), anemia 0.02% (95% CI: 0.02, 0.03), and hepatitis 0.01% (95% CI: 0.01, 0.01) (Data set 2). Among 42,325 severity cases, 152 cases resulted in death (0.36%).

### The prevalence of severe anemia between *P. vivax* and *P. falciparum*

**Fig. 5:**
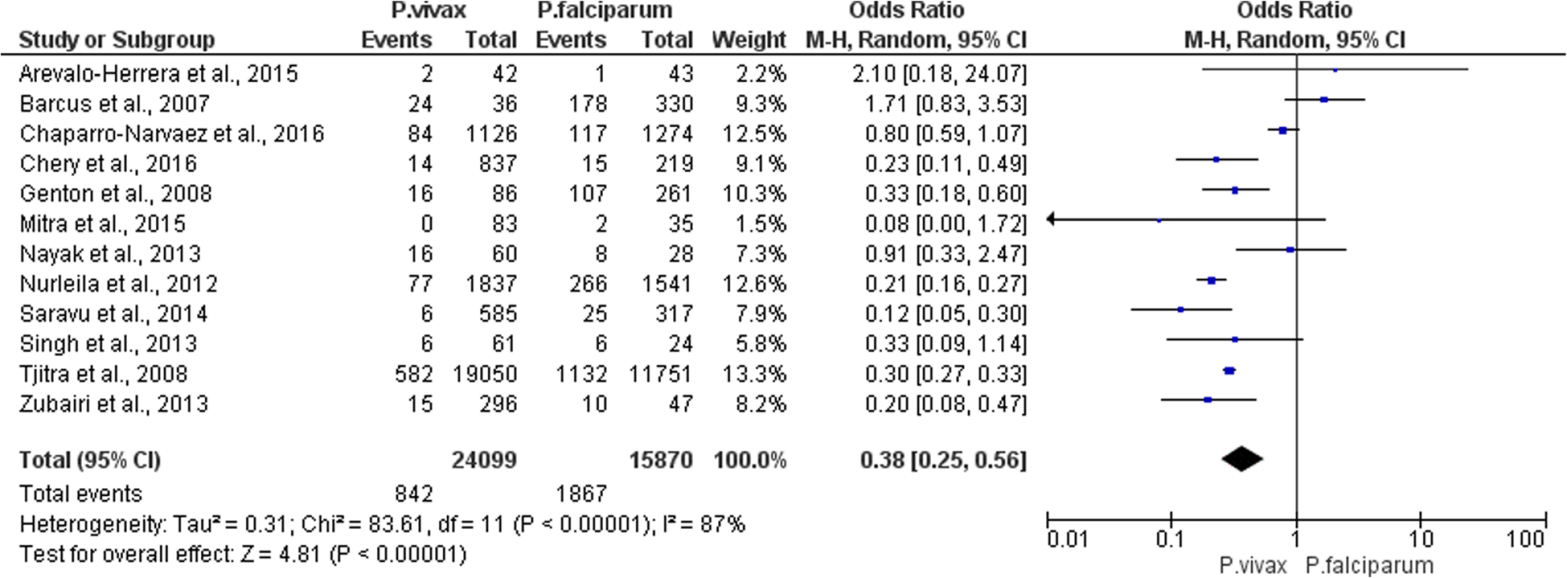
The prevalence of severe anemia between *P. vivax* and *P. falciparum*

There were 12 articles selected for comparing the prevalence of severe anemia between patients with *P. vivax* and *P. falciparum*. The meta-analysis showed that severe anemia was less frequent in patients with *P. vivax* compared to those with *P. falciparum* (*P*-value < 0.00001, OR = 0.38, 95%CI = 0.25–0.56, I^2^ = 87%) (Fig. 5). Considering each included study, none of these studies reported a higher incidence of severe anemia than those with *P. falciparum*.

### The prevalence of thrombocytopenia between *P. vivax* and *P. falciparum*

There were 6 articles selected for comparing the prevalence of thrombocytpenia between patients with *P. vivax* and *P. falciparum*. The subgroup analysis showed a non-significant difference in thrombocytopenia among *P. vivax* and *P. falciparum* (*P*-value = 0.23, I^2^ = 87%). Considering each included study, none of these studies reported a difference in thrombocytopenia among *P. vivax* and *P. falciparum*.

### Analysis of case reports

The association between patient characteristics and patient outcomes are shown in Table 3. Patients who developed impaired consciousness had well response (Discharged ≤ 7 days) when compared to other complications (*P*-value = 0.001, 95% CI = 0.14 (0.04–0.46)). Patients who developed convulsion had poor response (Discharged > 7 days) when compared to other complications (*P*-value < 0.0001, 95% CI = 3.93 (2.15–6.18)). Patients who developed respiratory distress had poor response (Discharged > 7 days) when compared to other complications (*P*-value = 0.009, 95% CI = 5.47 (1.5–21.39)). Patients who developed renal failure had poor response (Discharged > 7 days) when compared to other complications (*P*-value = 0.008, 95% CI = 7.08 (1.46–34.4)). Patients who developed jaundice had poor response (Discharged > 7 days) when compared to other complications (*P*-value = 0.021, 95% CI = 8.69 (1.05–72.1)). Patients who developed urine (anuria/oliguria) had poor response (Discharged > 7 days) when compared to other complications (*P*-value = 0.021, 95% CI = 8.69 (1.05–72.1)). Patients who developed splenomegaly at presentation had well response (Discharged ≤7 days) when compared to other complications (*P*-value = 0.009, 95% CI = 0.23 (0.08–0.72)). Patients who developed complications after the first anti-malarial drugs had poor response (Discharged > 7 days) when compared to other complications (*P*-value = 0.027, 95% CI = 3.35 (1.12–10)). The results also showed that patients who developed complications after the first drug regimen significantly required a second drug regimen (*P*-value = 0.001, 95% CI = 9.57 (1.95–46.98)) (Data set 3).

**Table 3:** Association between patient characteristics and days of well response

Patient Characteristics	Days of well response		***P***-value*	Odd’s ratio (95%CI)
	≥ 7 days	< 7 days		
**Impaired consciousness**				
- Yes	7 (17.1)	13 (59.1)	0.001	0.14 (0.04–0.46)
- No	34 (82.9)	9 (40.9)		
**Convulsion**				
- Yes	0	8 (4.5)	< 0.0001	3.93 (2.15–6.18)
- No	41 (100)	14 (99.5)		
**ARDS**				
- Yes	19 (46.3)	3 (13.6)	0.009	5.47 (1.5–21.39)
- No	22 (53.7)	19 (86.4)		
**Renal failure**				
- Yes	17 (41.5)	2 (9.1)	0.008	7.08 (1.46–34.4)
- No	24 (58.5)	20 (90.9)		
**Jaundice**				
- Yes	12 (29.3)	1 (4.5)	0.021	8.69 (1.05–72.1)
- No	29 (70.7)	21 (95.5)		
**Urine (anuria/oliguria)**				
- Yes	12 (29.3)	1 (4.5)	0.021	8.69 (1.05–72.1)
- No	29 (70.7)	21 (95.5)		
**Splenomegaly**				
- Yes	9 (22)	12 (54.5)	0.009	0.23 (0.08–0.72)
- No	32 (78)	10 (45.5)		
**Complications during treatment**				
- Yes	25 (61)	7 (31.8)	0.027	3.35 (1.12–10)
- No	16 (39)	15 (68.2)		
	**Required 2**^**nd**^**antimalarial drugs**			
	Yes	No		
**Complications during treatment**				
- Yes	11 (84.6)	23 (36.5)	0.001	9.57 (1.95–46.98)
- No	2 (15.4)	40 (63.5)		

*Pearson Chi-Square

The association between patient characteristics and time point of severe manifestation is demonstrated in Table 4. The results showed that respiratory distress was associated with the time point of severe manifestation (*P*-value = 0.002). Among 22 patients present with respiratory distress, 8 (36.4%) patients developed respiratory distress before treatment, 9 (40.9%) patients developed respiratory distress after treatment with anti-malarial drugs, and 5 (22.7%) patients developed respiratory distress before and after treatment. Among 28 patients present with renal failure, 22 (78.6%) patients developed renal failure before treatment, 1 (3.6%) patient developed renal failure before treatment, and 5 (17.9%) patients developed renal failure before and after treatment (*P*-value = 0.016). Among 8 patients developed bleeding, 2 (25%) developed bleeding before treatment, 2 (25%) patients developed bleeding before treatment, and 4 (50%) patients developed bleeding before and after treatment (*P*-value = 0.016). Among 12 patients present with headache, 4 (33.3%) patients developed complications before treatment, 6 (28%) patients developed complications after treatment, and 2 (16.7%) patients developed complications before and after treatment (*P*-value = 0.016). Among 7 patients present with rash, 4 (57.1%) patients developed complications before treatment, no patients developed complications before treatment, and 3 (42.9%) patients developed complications before and after treatment (*P*-value = 0.032). Among 19 patients with urine output abnormality, 15 (78.9%) patients developed complications before treatment, no patients developed complications before treatment, and 4 (21.1%) patients developed complications before and after treatment (*P*-value = 0.027).

**Table 4:** Association between patient characteristics and time of severe manifestation

Patient Characteristics	Time of severe manifestation			***P***-value*
	Before treatment	After treatment	Before and after treatment	
**ARDS**				
- Yes	8 (36.4)	9 (40.9)	5 (22.7)	0.002
- No	42 (77.8)	7 (13)	5 (9.3)	
**Renal failure**				
- Yes	22 (78.6)	1 (3.6)	5 (17.9)	0.016
- No	28 (58.3)	15 (31.2)	5 (10.4)	
**Bleeding**				
- Yes	2 (25)	2 (25)	4 (50)	0.003
- No	48 (70.6)	14 (20.6)	6 (8.8)	
**Headache**				
- Yes	4 (33.3)	6 (50)	2 (16.7)	0.018
- No	46 (71.9)	10 (15.6)	8 (12.5)	
**Rash**				
- Yes	4 (57.1)	0	3 (42.9)	0.032
- No	46 (66.7)	16 (23.2)	10 (10.1)	
**Urine (anuria/oliguria)**				
- Yes	15 (78.9)	0	4 (21.1)	0.027
- No	35 (61.4)	16 (28.1)	6 (10.5)	

*Pearson Chi-Square

Differences in patient characteristics and days of response are demonstrated in Table 5. The results showed that patients with poor response had a greater mean days of fever before presentation (mean = 6.05) than those with well response (mean = 4.78, *P*-value = 0.04). The results also showed that patients with poor response had more mean pulse rate (mean = 114.9) than those with well response (mean = 101.2, *P*-value = 0.04). Multivariate regression analysis demonstrated no association between patients with poor response and mean pulse rate after the multivariate regression analysis was performed by using age as a covariate (*P*-value = 0.057).

**Table 5:** Differences in patient characteristics and days of response

Parameters	Days of response, mean ± SD (N)		***P***-value*
	≥7 days	< 7 days	
Age	29.4 ± 11.5 (41)	27.1 ± 16.7 (21)	0.6
Days of fever	6.05 ± 2.58 (37)	4.78 ± 2.89 (22)	0.04
Pulse (per minute)	114.9 ± 20 (32)	101.2 ± 18.9 (13)	0.04
Temperature (°C)	38.5 ± 1.08 (26)	38.4 ± 1.04 (8)	0.76
Respiratory rate (per minute)	33.1 ± 12.6 (18)	26 ± 8.74 (10)	0.08
Systolic blood pressure (mmHg)	102.8 ± 21.2 (35)	103.2 ± 22.9 (15)	0.69
Diastolic blood pressure (mmHg)	65.4 ± 12.3 (34)	67.8 ± 16 (15)	0.28
Hemoglobin (g/dL)	10.1 ± 2.79	9.78 ± 2.67	0.69
Leukocyte count (× 10^3^ cell/μL)	6421.6 ± 2767.4 (19)	5900 ± 2943.9 (10)	0.61
Platelet count (×10^3^ cell/μL)	92,300 ± 85,080 (10)	50,700 ± 38,496 (3)	0.45

## Discussion

This analysis showed a large increase in publication on severe vivax malaria from year 1998–2013 with a maximum number of publications found in year 2013 (14, 28.6%). The numbers of publication were continuously decreased from year 2014–2018. That fluctuation seems likely due to underreporting or low number of severe vivax cases found in the last century, and only few data to support the dangers of *P. vivax* infections. Plasmodium species remained tagged with the term ‘benign’ in the last century. However, in recent years which peaked in 2013, a number of studies conducted in India [29–37], Sudan [38], Ethiopia [39], Korea [40], Brazil [41], and Pakistan [42] suggested a stronger association between *P. vivax* infections and severe manifestations, so that the number of publications on severe vivax and death were recognized than in previous centuries. The geographic distribution and the incidence of severe manifestations of vivax malaria were very specific and frequently found in some areas of the world. About half of these studies came from India (25, 51%) and also from other countries including Brazil, Colombia, Ethiopia, Indonesia, Korea, Malaysia, and Pakistan. This is very interesting because no prevalence of severe vivax malaria has been reported in other endemic regions such as Thailand, Cambodia, Lao, Burma, and Vietnam. However, a few case reports of severe vivax malaria have been found in Thailand [18]. In these reports, patients developed pulmonary edema after being treated with anti-malarial drugs.

The highest number of severity cases (629 cases) was found at Rumah Sakit Mitra Masyarakat (RSMM) hospital during 2004–2007 in India [43]. The pooled prevalence of the present study indicated that severe anemia, jaundice, respiratory distress, impaired consciousness, and acute renal failure were the most common complications found among 42,325 severity cases. Our results were in contrast to a previous systematic review indicating that severe thrombocytopenia (< 50,000/mm^3^) was the most common severe manifestation of severe vivax malaria at the beginning of year 2014 [44]. This might be due to the selection of articles in the study, whereas we selected more updated prevalence publications than their study with 19 studies in year 2014–2019. The present study found a large number of severe malarial anemia (hemoglobin< 5 g/dl) among patients with severe vivax malaria (87%) which was higher than among patients with severe falciparum malaria (73%) [43]. The severe anemia suggested that the death of infected reticulocytes led to severe anemia by stopping the supply of mature red blood cells [45, 46]. A previous study indicated that *P. vivax* malaria carries a well-known risk of splenic rupture, considered greater than for falciparum malaria thereby increasing the risk of severe malaria in vivax malarial patients [47]. Our meta-analysis showed that severe anemia occurred less frequently in patients with *P. vivax* compared to those with *P. falciparum*. It is believed that this is due to *P. falciparum* with a higher parasitemia causing a rapid drop in the hemoglobin level resulting in severe anemia [48]. Moreover, the severe anemia caused by *P. vivax* infection was due to the accumulation of smaller hemoglobin decrements with each recurrent infection [48].

In this study, minor complications of severe malaria which were not based on the WHO criteria were also demonstrated. The results indicated that thrombocytopenia and severe thrombocytopenia was the most common minor complications. Thrombocytopenia in patients with *P. vivax* and *P. falciparum* infection were the most common malaria-associated hematological complications [49]. However, the mechanisms are still unknown. Several mechanisms have been proposed to explain malaria thrombocytopenia. Some studies suggested that thrombocytopenia in malaria might be caused by platelet activation, consumption [50], and platelet apoptosis [28] which leads to destruction by the immune system [51]. Our results were in accordance with a previous systematic review indicating that 10.1% of patients with *P. vivax* had severe to very severe thrombocytopenia [52]. Moreover, one study indicated that patients with severe *P. vivax* had a 2.8 -fold higher risk of developing severe thrombocytopenia than those with severe *P. falciparum* malaria [53].

Analysis from the case reports showed that patients who developed impaired consciousness and splenomegaly had well response (Discharged ≤ 7 days) whereas patients who developed convulsion, respiratory distress, renal failure, urine (anuria/oliguria), jaundice, and complications after being treated with the first anti-malarial drugs had poor response (Discharged > 7 days). A previous study demonstrated splenomegaly and altered consciousness were more frequent in cases of severe vivax malaria and suggested that these symptoms could indicate a poor prognosis of malaria [54]. For respiratory distress, a previous study indicated that treatment of *P. vivax* resulted in initial worsening of airflow obstruction and gas transfer, which took 6 hours to 8 days after the initiation of anti-malarial treatment [55]. Patients who develop respiratory distress required the mechanical ventilation and other advanced life-support strategies, were delayed in receiving a diagnosis and efficient treatment which can lead to mortality [56]. The results also demonstrated that respiratory distress was associated with the time point of severe manifestations. The present study indicated that 8 patients (36.4%) developed respiratory distress before treatment and 9 patients (40.9%) developed respiratory distress after treatment with anti-malarial drugs. In patients with post- respiratory distress, it is suggested that inflammatory reaction occurred within lung microstructure after treatment with anti-malarial drugs was the cause of the in respiratory impairment [55, 57]. It is also needs to be recognized that convulsion in *P. vivax* infection may lead to poor outcome and increased mortality in *P. vivax* malaria. For renal failure, treatment of malaria-associated kidney diseases required a long-term duration due to management of appropriate antimalarial drugs, hydroelectrolytic disturbance corrections, fluid replacement, and dialysis [58]. A previous study indicated that early initiation of dialysis for acute renal failure was associated with good outcomes by reducing case fatality and increasing renal recovery [59]. In our study, most of the patients developed renal failure before treated with anti-malarial drugs (22, 78.6%). A previous study indicated that *P. vivax* malaria can cause renal failure more commonly than *P. falciparum* malaria, where renal ischemia was the dominant pathogenic mechanism [60].

According to the WHO criteria, jaundice is one of the cardinal manifestations of severe malaria in adults [2]. This study indicated that patients with jaundice had poor response to treatment (Discharged > 7 days). Jaundice in malaria is caused by destruction of parasitized RBCs leading to massive intravascular hemolysis, resulting in hepatic dysfunction and severe anemia [61, 62]. A previous study indicated that treatment of *P. vivax* with IV artesunate rapidly reduced parasitemia levels; however, patients underwent post-treatment hemolysis with the higher doses of IV artesunate [63]. This post-treatment hemolysis may induce jaundice, hemoglobinuria, hepatomegaly, or severe malaria after treatment with the first anti-malarial drugs. However, no higher doses of IV artesunate had been reported among 76 cases.

This study also showed that patients with poor response had more mean days of fever before presentation (mean = 6.05) than those with well response (mean = 4.78, *P*-value = 0.04). Fever in malaria is believed to be associated with release of toxins and antigenic substances, which induce the release of cytokines by white blood cells. Our study results indicated poor prognosis of patients who had more days of fever before presentation. During treatment, fever in severe malaria usually resolves by Day 4 to 5 and needs prompt investigations in case of a prolonged fever after severe malaria [64]. To our knowledge, the prolonged fever before presentation was caused by the human body tries to clear parasites for a period of time until patients present at the hospital. Although the significant difference between days of fever and poor response existed, this information should be carefully interpreted and needs to be confirmed by further studies with a greater sample size to gain significant data on daily practice.

The results of the present study also showed that patients with poor response had a greater mean pulse rate than those with well response. We thought it could be cardiovascular involvement related to the previous studies, indicating that *P. vivax* infection is associated with rare and serious cardiovascular complications [65]. Although the results of greater mean pulse rate were related to poor outcome of patients, there were other variables such as fever that could be confounding factors. From the present data, the multivariate analysis of fever as a covariate could not be performed because most patients in the case reports were presented with fever at presentation (74/76, 97.4%). This analysis needs to be confirmed by further studies.

### Limitations

The present study had a main limitation in that we could not retrieve all full-text and non-English articles that passed our inclusion criteria.

## Conclusion

Severe anemia was the most common major manifestation of *P. vivax* malaria. Severe anemia was found less frequently in patients with *P. vivax* than those with *P. falciparum*. Renal failure, jaundice, anuria/oliguria, and complication during treatment along with mean days of fever and higher pulse rates at presentation might be predictors of poor outcome of patients with severe vivax malaria.

## Supplementary information

**Additional file 1.** Data set 1.

**Additional file 2.** Data set 2.

**Additional file 3.** Data set 3.

## Data Availability

All data generated or analyzed during this study are included in this manuscript and its supplementary information files**.**

## Abbreviations

ISI: Web of Science
WHO: World Health Organization
